# Lack of Modulation of Nicotinic Acetylcholine Alpha-7 Receptor Currents by Kynurenic Acid in Adult Hippocampal Interneurons

**DOI:** 10.1371/journal.pone.0041108

**Published:** 2012-07-25

**Authors:** Peter Dobelis, Kevin J. Staley, Donald C. Cooper

**Affiliations:** 1 Center for Neuroscience, Institute for Behavioral Genetics, Department of Neuroscience, University of Colorado at Boulder, Boulder, Colorado, United States of America; 2 Department of Neurology, Massachusetts General Hospital, Boston, Massachusetts, United States of America; Neuroscience Campus Amsterdam, VU University, Netherlands

## Abstract

Kynurenic acid (KYNA), a classical ionotropic glutamate receptor antagonist is also purported to block the α7-subtype nicotinic acetylcholine receptor (α7* nAChR). Although many published studies cite this potential effect, few have studied it directly. In this study, the α7*-selective agonist, choline, was pressure-applied to interneurons in hippocampal subregions, CA1 stratum radiatum and hilus of acute brain hippocampal slices from adolescent to adult mice and adolescent rats. Stable α7* mediated whole-cell currents were measured using voltage-clamp at physiological temperatures. The effects of bath applied KYNA on spontaneous glutamatergic excitatory postsynaptic potentials (sEPSC) as well as choline-evoked α7* currents were determined. In mouse hilar interneurons, KYNA totally blocked sEPSC whole-cell currents in a rapid and reversible manner, but had no effect on choline-evoked α7* whole-cell currents. To determine if this lack of KYNA effect on α7* function was due to regional and/or species differences in α7* nAChRs, the effects of KYNA on choline-evoked α7* whole-cell currents in mouse and rat stratum radiatum interneurons were tested. KYNA had no effect on either mouse or rat stratum radiatum interneuron choline-evoked α7* whole-cell currents. Finally, to test whether the lack of effect of KYNA was due to unlikely slow kinetics of KYNA interactions with α7* nAChRs, recordings of a7*-mediated currents were made from slices that were prepared and stored in the presence of 1 mM KYNA (>90 minutes exposure). Under these conditions, KYNA had no measurable effect on α7* nAChR function. The results show that despite KYNA-mediated blockade of glutamatergic sEPSCs, two types of hippocampal interneurons that express choline-evoked α7* nAChR currents fail to show any degree of modulation by KYNA. Our results indicate that under our experimental conditions, which produced complete KYNA-mediated blockade of sEPSCs, claims of KYNA effects on choline-evoked α7* nAChR function should be made with caution.

## Introduction

Nicotinic acetylcholine receptors (nAChRs) are ligand-gated, nonselective cation channels. To date, nine α-subunits (α2–10) and three β-subunits (β2–4) have been discovered in the CNS (Reviewed in [1], [2], [3]). The α-subunits are required for ligand activation while the β-subunits serve as structural components and can affect receptor characteristics, such as ligand affinity and desensitization rate [1], [2], [3]. Heterologous expression studies, as well as studies with null mutant mice show that these subunits assemble in various combinations to form pharmacologically and biophysically distinct nAChR subtypes and these subtypes show regionally distinct patterns of expression [1], [2], [3].

In the hippocampus, at least three nAChR subtypes are expressed: those containing the α4 and β2 subunits (α4β2*, the * indicating the possibility of other subunits [4]), those composed of α7 subunits (α7*) and those possibly containing the β4 subunit containing receptors (putatively α3β4*) [5], [6], [7], [8], [9], [10], [11], [12], [13]. Previous studies demonstrated that the α4β2* receptors are located on GABAergic cell bodies and nerve terminals [11], [12], [14], [15]. The α7* receptors are located on some GABAergic soma and at least some glutamatergic nerve terminals [5], [6], [9], [11], [12], [16]. The putative β4 containing receptors appear to be associated with some glutamatergic activity [13], but their precise localization remains to be determined.

Kynurenic acid (KYNA) is a well-established antagonist of the AMPA-,NMDA-, and kainite-type glutamate receptors [17], [18]. A metabolite of tryptophan, KYNA is synthesized primarily by glia and released into the extracellular space (Reviewed in [18], [19]). Although cerebral spinal fluid (CSF) levels of KYNA are below the established IC_50_ values for AMPA and NMDA receptors, some studies indicate de novo synthesis and release of KYNA reduces glutamate-mediated excitotoxicity suggesting that KYNA release may be located near synaptic sites thus creating micro domains of high KYNA concentration [19], [20].

In 2001, it was reported KYNA also blocks α7* nAChRs [21]. This study measured the direct effects of KYNA on α7* receptors expressed in cultured embryonic hippocampal neurons and revealed that KYNA had greater affinity for α7* receptors than for NMDA receptors [21]. Additional studies in hippocampal slices showed that KYNA reduced choline-evoked increases in GABAergic spontaneous inhibitory postsynaptic currents (sIPSCs); an indirect measure α7* function. However, the KYNA effect in slices was much less robust than that seen in cultured neurons [21]. The lower potency of KYNA for α7* receptors in hippocampal slices as compared to cultured neurons was interpreted to result from diffusion barriers inherent to slices as well as the relative hydrophobicity of KYNA (however, a recent report suggest that the age of the tissue could account for the reduced effects of KYNA [26]). Subsequent studies directly measured the effects of KYNA on α7* nAChRs expressed in hippocampal slices confirming the results of their initial report. Recently, however, reports have failed to find any effect of KYNA on α7*-mediated events and we present further support for the lack of KYNA effects on α7* nAChRs currents using direct patch-clamp recording from adolescent or mature rodent acute brain slices. [22], [23].

## Materials and Methods

### Hippocampal Slices

Male C57BL/6J/Ibg mice, 45- to 60-days old, were obtained from the Institute for Behavioral Genetics (Boulder, CO). Male Sprague Dawley rats, 21–28 days old, were obtained from **(**Harlan, Wilmington, MA) and tested at 30–45 days of age. Housing and treatment of all animals were in accordance with the NIH and the University of Colorado, Boulder IACUC guidelines. The mice were sacrificed by cervical dislocation and rats were sacrificed by isoflurane anesthesia. The brains were removed quickly and placed into a “cutting solution” of the following composition (in mM): Sucrose 75, NaCl 87, NaHCO_3_ 25, KCl 2.5, NaH_2_PO_4_ 1.25, 0.5 CaCl_2_ MgCl_2_ 7, and glucose 25, bubbled continuously with a mixture of 95% O_2_ and 5% CO_2_ at 4°C. The brains were blocked and secured to the cutting platform using cyanoacrylate glue. Horizontal hippocampal slices (250 µm thickness for mice, 300 µm thickness for rat) were obtained using a Vibratome (VT1000P, Leica Microsystems, Wetzlar, Germany) and transferred to a storage chamber containing a continuously bubbled solution comprised of a 50∶50 mixture of cutting solution and artificial CSF (ACSF) of the following composition (in mM): NaCl 126, NaHCO_3_ 26, KCl 3, NaH_2_PO_4_ 1.2, CaCl_2_ * 2H_2_O 2.4, MgCl_2_ 1.5, and glucose 10. Slices were allowed to equilibrate for at least 1 hr at 34–35°C before they were transferred to the recording chamber.

### Electrophysiological Recordings

All experiments were performed at 32–34°C while the tissue was superfused with ACSF at a rate of 2.5 ml/min. Whole-cell patch-clamp recording was accomplished by using glass pipettes pulled on a Flaming/Brown electrode puller (Sutter Instruments, Novato, CA). The resistance of the pipettes was 3–5 MΩ when filled with a potassium gluconate-based internal solution, which consisted of (in mM): 132 K-Gluconate, 4 KCl, 1 EGTA, 2 MgCl2, 0.1 CaCl2, 2 Mg-ATP, 0.3 Na-GTP, and 10 HEPES adjusted to pH 7.25 with additional KOH. Cells were viewed with an upright microscope equipped with IR-DIC optics (Nikon 800 FN, or Olympus BX51WI). Neurons were recorded from using the whole-cell voltage clamp technique with a Multiclamp 700 (Axon Instruments, Foster City, CA). Data were recorded to a desktop computer and analyzed off-line using pClamp 9 software (Axon Instruments, Foster City, CA).

### Drug Application

Kynurenic acid, DHβE, and MLA were delivered by bath application. Brief pulses (10–300 ms) of choline (10 mM) were applied directly to the cell body via pressure microejection (2–10 psi, pipette tip ≈20–50 µm from cell border) from pipettes identical to the recording pipettes, using a Picospritzer II (General Valve, Fairfield, NJ). Due to the brief duration of agonist application (10–300 ms), choline was applied at 20–30 second intervals without any measurable desensitization. Glutamate was applied in a similar fashion (2–10 psi, 10–100 ms) but the interval between puffs was extended to five minutes to avoid possibility of glutamate receptor-induced plasticity effects on the glutamate-evoked and spontaneous synaptic glutamate currents.

### Drugs Used

Methyllycaconitine (MLA) citrate, dihydro-β-erythroidine (DHβE), kynurenic acid (KYNA), choline, and glutamate were purchased from Sigma (St. Louis, MO).

### Statistical Analysis

Data were analyzed using either the paired or unpaired student’s t-test, where appropriate.

## Results

### Kynurenic Acid Effects on α7* nAChRs Expressed on Mouse Hilar Interneurons

Previous studies of rat hilar neurons revealed functional α7* nAChRs [24], and studies in the mouse revealed a high density of α7* nAChRs using a ligand binding assay [25]. We sought to investigate whether mouse hilar interneurons expressed functional a7* nAChRs.

**Figure 1:**
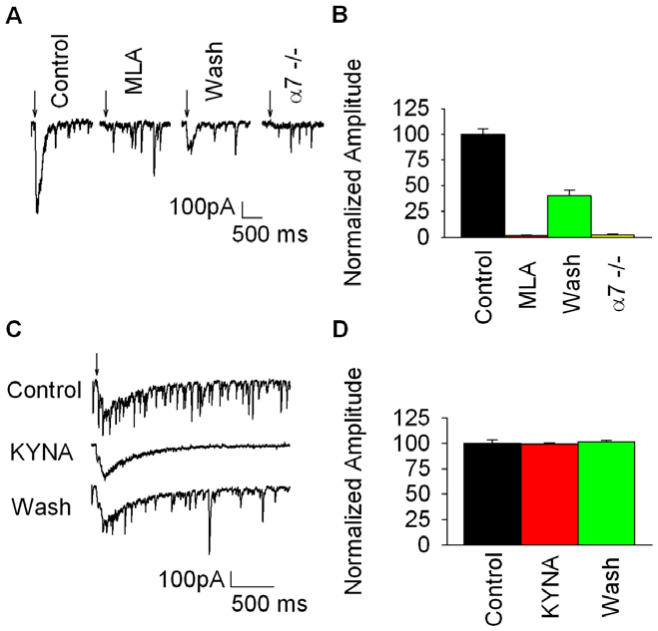
KYNA effects on α7* currents in mouse hilar interneurons. Panel A shows representative traces for the characterization of α7* currents in mouse hilar interneurons. The left most trace shows the control response to pressure applied choline. The next two traces show the response to choline in the presence of MLA (10 nM) and after 30 min washout, respectively. The last trace shows the lack of response to choline in α7 null mutant mice. Panel B shows the summarized data for these experiments. MLA blocked completely the choline response (t = 6.6153, df = 7, p = 0.0003, n = 8, paired t-test, control vs. MLA,), the MLA effect was reversed partially after washout (t = 2.6083, df = 7, p = 0.035, n = 8, paired t-test washout vs. MLA; t = 4.029, df = 7, p = 0.005, n = 8, paired t-test washout vs. control,), and the choline response for the α7 null mutant mice differed significantly from wild type (t = 4.7682, df = 14, p = 0.0003, n = 8, unpaired t-test,). Panel C shows representative traces for the effect of bath applied KYNA on choline-evoked α7* currents. The top trace shows the control response to pressure applied choline; note the overriding glutamatergic spontaneous EPSCs. The middle trace shows the choline response after 30 min exposure to 1 mM KYNA; note the absence of the spontaneous EPSCs. The bottom trace shows the response to choline after 20 min washout of KYNA; note the reappearance of the spontaneous EPSCs. Panel D shows the summarized results for these experiments. KYNA failed to produce any reduction in the choline response (t = 0.0381, df = 19, p = 0.97, n = 20).

The present study utilized pressure application of choline (10 mM) to adolescent-to-adult hilar neurons under voltage-clamp control. The initial results showed that choline application elicited an inward current that was completely blocked by the bath application of the α7-selective antagonist, MLA (Figure 1A and 1B). As an additional control we performed experiments using α7 null mutant mice that revealed no choline-evoked responses under identical experimental conditions (Figure 1A and 1B). Together, these results indicate that the choline-evoked inward currents recorded from mouse hilar interneurons were mediated by functional α7* nAChRs. To the best of our knowledge, this is the first demonstration of functional a7* nAChRs expressed in mouse hilar region.

One characteristic of hilar interneuron recordings is the high frequency, large amplitude spontaneous excitatory postsynaptic currents (sEPSCs) that are resistant to MLA and made the analysis of the a7* currents problematic (see Figure 1A 1C). To determine the pharmacology of these sEPSCs, we applied a saturating concentration (1 mM) of the broad spectrum ionotropic glutamate receptor antagonist KYNA. Bath applied KYNA (1 mM) completely blocked the sEPSCs, indicating the sEPSCs were glutamatergic. Surprisingly, concurrent measurements of evoked α7* currents revealed that 1 mM KYNA failed to block these responses (Figure 1C and 1D) indicating that the a7* currents in mouse hilar interneurons were insensitive to KYNA. Figure 1C shows representative choline-evoked α7* currents before, during, and after bath applied KYNA (1 mM). Notice that while the sEPSCs are absent during the presence of KYNA, the α7* current is unaffected. Out of 23 neurons studied, 20 displayed choline-induced and KYNA resistant whole-cell currents; the remaining three neurons were unresponsive to choline. The results presented here showed that all of the choline responsive mouse hilar neurons fail to show evidence of modulation by KYNA.

### Effects of KYNA on Glutamatergic Whole-cell Currents in Mouse Hilar Interneurons

**Figure 2:**
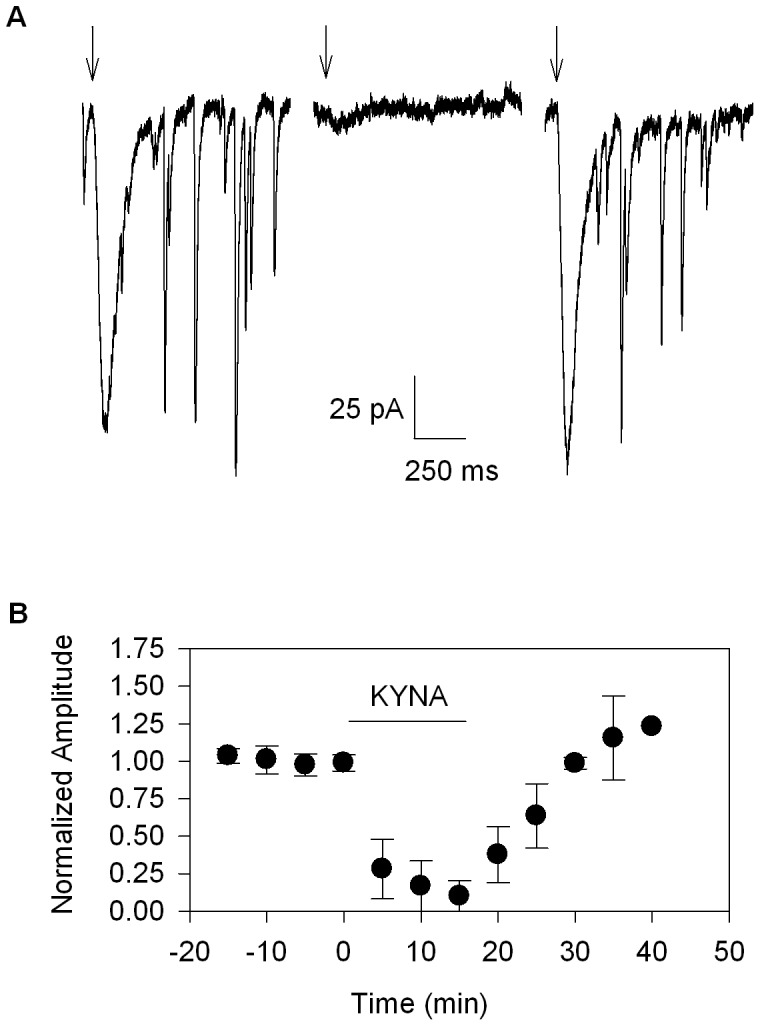
KYNA effects on exogenously applied glutamate-induced whole-cell currents. Panel A shows representative traces for whole-cell currents evoked by pressure-applied glutamate (arrows; 100 µM glutamate, 2–10 psi, 10–100 ms). Panel B shows the combined results (t = 8.8502, df = 4, p<0.001, n = 5, paired t-test).

In the original report of KYNA blockade of α7* nAChRs, the effect of KYNA of α7* nAChRs was much less pronounced in acute hippocampal slices compared to cultured hippocampal neurons [21]. The authors concluded that the reduced effect of KYNA on α7* nAChR function in slices was due to a reduced ability of KYNA to penetrate the hippocampal slice preparation. This is unlikely, given that KYNA readily blocked sEPSCs in the current study, while having no effect on the α7* currents. However, one possible explanation for the lack of KYNA blockade of α7* current in the current study is that the pressure application of choline displaced KYNA from its binding site. To test for this possibility, we pressure-applied glutamate and determined the effects of bath-applied KYNA on glutamate-evoked whole-cell currents in mouse hilar interneurons. Our results showed that pressure-applied glutamate failed to displace KYNA from its binding site and completely blocked both the exogenous glutamate currents and the endogenous sEPSCs. Furthermore, the onset of antagonism was rapid, with substantial block after 15 min. bath exposure, and complete reversal of blockade after 20 min. washout (Figure 2). The results of these control experiments address two main technical questions related to the lack of modulation of α7* nAChR current by KYNA indicating that minimal diffusion barriers exist for KYNA in the hippocampal slice preparation, and that pressure-applied agonist does not displace KYNA from its site of action. Given that KYNA has a greater affinity for α7* nAChRs compared to glutamate receptors [21], we interpret that the lack of KYNA blockade of α7* currents in the current study is not best explained by its displacement by the pressure application of choline.

### KYNA Effects on CA1 α7* nAChRs in Mouse and Rat Stratum Radiatum Interneurons

**Figure 3:**
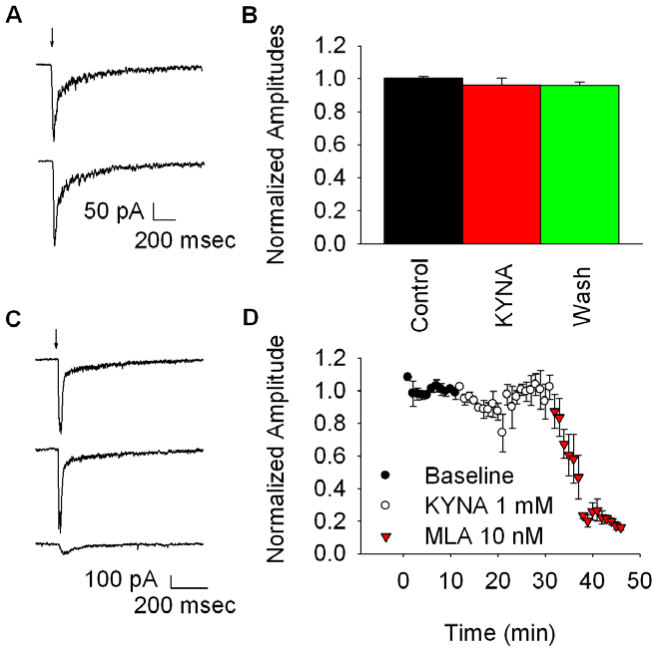
KYNA effects on α7* responses in mouse and rat CA1 stratum radiatum interneurons. Panel A shows representative traces for choline-evoked α7* currents in mouse stratum radiatum interneurons. The top trace is the control response to pressure applied choline (10 mM, 50 ms). The bottom trace shows the choline response after 30 min bath exposure to 1 mM KYNA. Panel B shows the summarized results. KYNA failed to reduce the choline response (t = 0.8565, df = 4, p = 0.44, n = 5, paired t-test). Panel C shows representative traces for pressure applied choline-evoked α7* currents in rat stratum radiatum interneurons. The top trace shows the control response to 10 mM choline. The middle trace shows the ACh response after 30 min bath exposure to 1 mM KYNA. The bottom trace shows the ACh response after 20 min bath exposure to 10 nM MLA. Panel D shows the summarized results for these experiments. For this analysis, the last five traces were averaged for each condition: control, KYNA, and MLA. KYNA failed to block the choline-induced α7* currents (t = 0.3994, df = 4, p = 0.71, n = 5, paired t-test) while MLA blocked significantly the response (t = 15.5441, df = 4, p = 0.0001, n = 5, paired t-test).

Previously published reports of KYNA effects on α7* nAChRs in hippocampal slices were done in interneurons located in the rat CA1 stratum radiatum subfield [21], [26], [27], [28]. Another possible explanation for the lack of KYNA effect on hilar α7* nAChRs is that they are somehow different from those expressed in the CA1 region; possibly due different post translational modification or the inclusion/exclusion of additional subunits. Indeed, evidence exists that native α7* nAChRs may include other subunits [29], [30], [31], [32], [33]. To test this hypothesis, we measured the KYNA sensitivity of choline-evoked α7* currents expressed in mouse CA1 stratum radiatum interneurons and again found no evidence for KYNA modulation of α7* nAChRs (Figure 3A and 3B).

Another possible explanation for the lack of KYNA blockade of mouse α7* nAChRs is that they differ from those expressed in the rat. Papke and colleagues showed that pharmacological differences exist between rat and human α7 nAChRs expressed in oocytes [34], [35]. We tested for this by recording choline-evoked α7* currents expressed in rat CA1 stratum radiatum interneurons, and again found no evidence for KYNA blockade (Figure 3C and 3D).

### Effects of Long Term KYNA Exposure on α7*-mediated Whole-cell Currents

**Figure 4:**
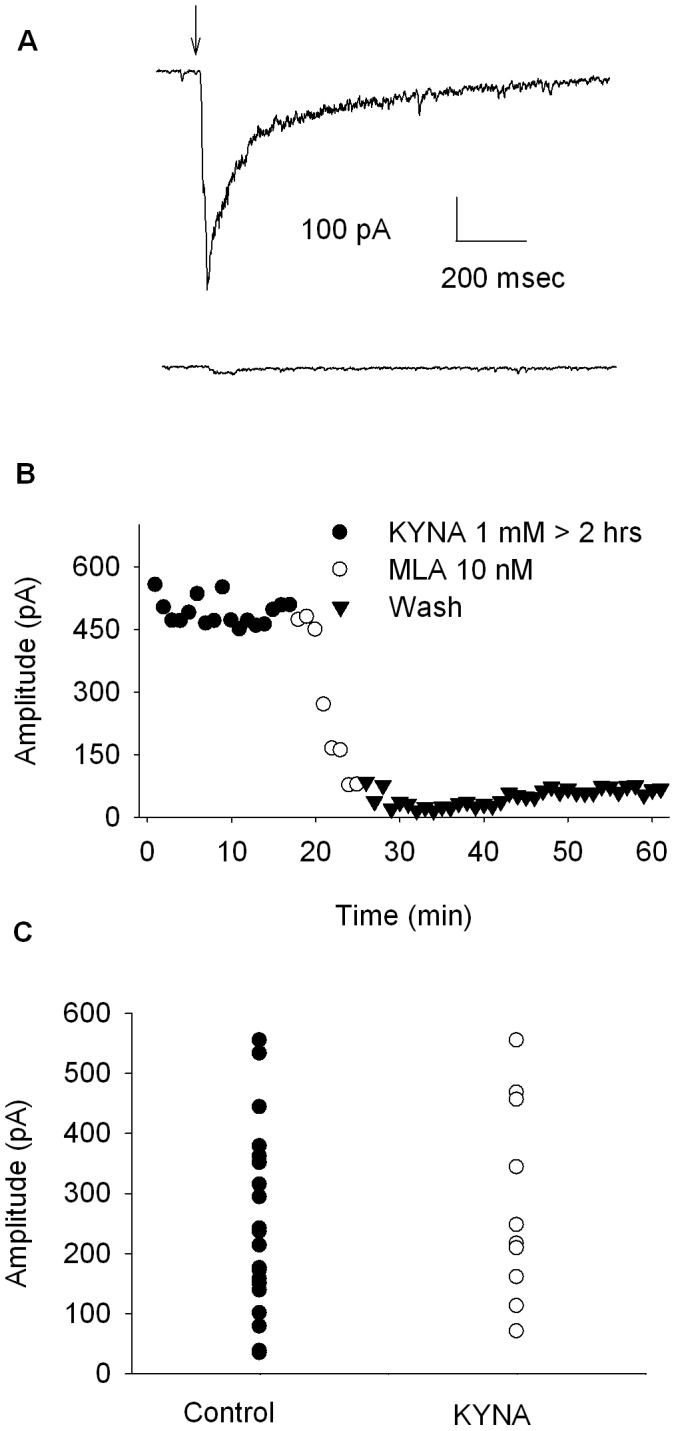
Effects of long term exposure to KYNA on pressure-applied choline-evoked α7* currents. In these experiments, slices were prepared, stored and recorded in the presence of 1 mM KYNA (at least 90 min exposure). Panel A shows representative traces for a baseline choline-evoked α7* current after 2 hrs exposure to KYNA (top trace) and after 5 min exposure to MLA (10 nM, bottom trace). Panel B shows the time course for this experiment. Panel C shows the distributions for control choline-evoked α7* currents (n = 20) and choline-evoked α7* currents after at least 90 min exposure to 1 mM KYNA (n = 10). Each point represents the average of 5–10 events, the error bars were omitted for clarity. There was no significant difference between groups (t = 0.5899, df = 28, p = 0.56, unpaired t-test).

Hilmas et al., (2001) [21] suggested that KYNA blockade of α7* nAChRs is slow to develop in the slice. To address this issue, experiments were done in which hippocampal slices were cut, stored, and continuously perfused with 1 mM KYNA. Additionally, 1 mM KYNA was present in the in the choline (10 mM) puffer pipette to control for the possibility that choline application was displacing KYNA from its site of action. In these experiments, the slices were exposed to 1 mM KYNA for at least 90 min. Figure 4A shows representative traces from a hilar neuron exposed to 1 mM KYNA for 2 hrs. The top trace shows the inward response to a 30 ms pressure application of 10 mM choline/1 mM KYNA. The bottom trace shows the choline response was blocked by 10 nM MLA, indicating that it was mediated by α7* nAChRs and not due to a mechanical artifact resulting from pressure application. Figure 4B presents the time course for this experiment, showing stable, large amplitude α7* nAChR-mediated currents in the presence of 1 mM KYNA that were subsequently blocked by bath applied MLA (10 nM).

Because no baseline responses were obtained in these experiments, the range of amplitudes of choline-evoked responses obtained in the absence of KYNA from separate experiments were compared to those obtained after at least 90 min of continuous KYNA exposure and are summarized in Figure 4C. The amplitudes of baseline choline-evoked responses ranged from 34–574 pA (n = 20). Choline-evoked responses from neurons exposed to KYNA for at least 90 min ranged from 185–557 pA (n = 10). Given the large range of amplitudes for each condition, no statistical significance for a KYNA effect was seen. However, if KYNA was partially blocking α7* receptors, one would expect to see shift in the range of amplitudes to the lower end, which was not observed. These results show that long-term KYNA exposure has no effect on choline-evoked α7*-mediated currents. Also, the inclusion of KYNA in the application pipette confirms, yet again that choline application in the previous experiments was not displacing KYNA from its supposed binding site on the receptor.

## Discussion

Results presented here failed to replicate prior reports by the Albuquerque laboratory [21], [26], [27], [28] showing that KYNA blocks α7* nAChRs. However, our negative results are consistent with those reported recently by the Hernandez-Guijo and Kew laboratories [22], [23].

In the original report of KYNA antagonism of α7* nAChRs, Hilmas et al., (2001) [21] stated that DMSO was used to get KYNA into solution. It is possible that high concentrations of DMSO necessary to dissolve KYNA produced indirect nonspecific effects. Indeed, one group that failed to observe a modulatory role for KYNA on α7* nAChRs, Mok et al., (2009) [23] showed that high concentrations of DMSO inhibit α7* currents regardless of the presence of KYNA. This result may explain the discrepancy between the initial report [21] and the results presented here, as well as those of Mok et al.,(2009) [23]. However, these discrepancies are not accounted for with later reports of KYNA effects on α7* nAChRs citing that KYNA was dissolved using NaOH [26], [27].

There is increasing evidence that some native α7* nAChRs may be heteromeric (i.e., containing non-α7 subunits [14], [29], [30], [31], [32], [33]. These studies show that heteromeric α7* nAChRs have different pharmacological and biophysical properties compared to homomeric α7 nAChRs. Since nAChR subunits are differentially expressed both regionally and developmentally [36], this raises the possibility that regional differences in α7* nAChR subunit composition could account for the differences in sensitivity to KYNA. To determine if our initial lack of KYNA effect on α7* currents was due to a regional difference in α7* nAChRs (i.e., hilar vs. CA1), we recorded choline-evoked α7* currents in CA1 stratum radiatum interneurons. These studies also revealed no effect of KYNA indicating that with regard to KYNA sensitivity, α7* nAChRs in the hilus and CA1 stratum radiatum are similar.

Species differences in pharmacological sensitivity of α7 nAChRs have been reported [34], [35] and could account for the lack of effect we saw in our initial studies of KYNA effects on mouse α7* nAChRs. To address this, we measured the ability of KYNA to block choline-evoked α7* currents in rat CA1 stratum radiatum interneurons and, again, found no effect. This result was also reported by Mok et al., (2009) [23].

One indirect measure that resulted in a positive modulatory action of KYNA on α7* nAChR function was choline-evoked GABA release in hippocampal slices [21], [23]. This phenomenon is action potential dependent [23] and, as such, requires the coordinated action of several cellular functions (i.e., activation of voltage-gated sodium and calcium channels) required for neurotransmitter release. Therefore, KYNA could be acting nonspecifically anywhere between the activation of the α7* nAChRs and the activation of GABA receptors. Indeed, Mok et al., (2009) [23], showed that KYNA blocked α7* nAChR-induced increases in GABAergic synaptic transmission, however, like the results we report here, concurrent recordings of α7* currents showed no effect of KYNA on these currents. These authors also showed that KYNA blocked GABA_A_ receptors in cultured rat hippocampal neurons, however, this result was not replicated for spontaneous GABAergic IPSCs in hippocampal slices. Together, these results indicate that in adolescent rat brain, KYNA blocks choline-evoked GABAergic synaptic transmission at a site other than the α7* or GABA_A_ receptors.

One explanation for the variability of KYNA effect on α7* nAChR function reported in the literature put forth by Albuquerque and colleagues [26] is the age of the preparation. They report that α7* nAChRs in preweaned (<18 days old) rat hippocampal slices are insensitive to KYNA, while α7* nAChRs in postweaned (>18 days old) rats are sensitive to KYNA blockade. On the surface this explanation seems plausible as both Arnaiz-Cot et al [22] and Mok et al., (2009) [23] report that α7* nAChRs expressed in cell culture are insensitive to KYNA blockade, however, Hilmas et al., (2001) [21] report the opposite. Additionally, the results presented here used tissue obtained from adolescent to adult mice (45–60 days old) and from early adolescent to adolescent rats (30–45 days old). Given the wide range of results for comparable preparations it seems more likely that subtle differences in methodology not discernible from the published methods are responsible for the disparate results.

One result that appears to be consistent is the effect of KYNA to block choline-induced increases in GABAergic function in hippocampal slices. Both Albuquerque and colleagues [21], [26], [28], [37] and Mok et al., (2009) [23] report that KYNA blocks choline-induced increases in GABAergic function in hippocampal slices. However, concomitant recordings of α7* nAChR function in these experiments revealed that this was not due to α7* nAChR blockade [23]. Recently, KYNA was demonstrated to be an agonist for the orphan g-protein receptor GPR-35 [38], [39]. GPR-35 is expressed in the brain [40] and is linked to the G_i-o_ pathway [38], [39]. Other receptors coupled to the G_i-o_ pathway have been shown to block action potential-dependent neurotransmitter release (i.e., the GABA_B_ receptor and metabotropic glutamate receptor groups II & III, Reviewed in: [41], [42], [43]). If GPR-35 is located on GABAergic nerve terminals, this raises the possibility that KYNA actions previously attributed to its effects on α7* nAChRs could be the result of its actions on GPR-35 or some other pharmacological target. Regardless, the results presented here, as well as the finding that KYNA could be acting through GPR-35, suggest that caution should be used when interpreting the mechanism of action of KYNA in complex preparations.
